# Influenza

**Published:** 1849-01

**Authors:** 


INFLUENZA.

Influenza is almost universal in Louisville at this time. It is difficult to meet with a person who is not laboring under the affection, or has not been troubled with it. The form, however, is unusually mild, few cases requiring medication. It will occur to the minds of our readers, that influenza spread over the continent the winter before cholera made its appearance upon our shores before. Still, we have had influenza several times since without cholera.

